# Swabbing Often Fails to Detect Amphibian Chytridiomycosis under Conditions of Low Infection Load

**DOI:** 10.1371/journal.pone.0111091

**Published:** 2014-10-21

**Authors:** Jaehyub Shin, Arnaud Bataille, Tiffany A. Kosch, Bruce Waldman

**Affiliations:** Laboratory of Behavioral and Population Ecology, School of Biological Sciences, Seoul National University, Seoul, South Korea; University of Minnesota, United States of America

## Abstract

The pathogenic chytrid fungus, *Batrachochytrium dendrobatidis* (denoted Bd), causes large-scale epizootics in naïve amphibian populations. Intervention strategies to rapidly respond to Bd incursions require sensitive and accurate diagnostic methods. Chytridiomycosis usually is assessed by quantitative polymerase chain reaction (qPCR) amplification of amphibian skin swabs. Results based on this method, however, sometimes yield inconsistent results on infection status and inaccurate scores of infection intensity. In Asia and other regions where amphibians typically bear low Bd loads, swab results are least reliable. We developed a Bd-sampling method that collects zoospores released by infected subjects into an aquatic medium. Bd DNA is extracted by filters and amplified by nested PCR. Using laboratory colonies and field populations of *Bombina orientalis*, we compare results with those obtained on the same subjects by qPCR of DNA extracted from swabs. Many subjects, despite being diagnosed as Bd-negative by conventional methods, released Bd zoospores into collection containers and thus must be considered infected. Infection loads determined from filtered water were at least 1000 times higher than those estimated from swabs. Subjects significantly varied in infection load, as they intermittently released zoospores, over a 5-day period. Thus, the method might be used to compare the infectivity of individuals and study the periodicity of zoospore release. Sampling methods based on water filtration can dramatically increase the capacity to accurately diagnose chytridiomycosis and contribute to a better understanding of the interactions between Bd and its hosts.

## Introduction

The emerging infectious disease chytridiomycosis causes morbidity and mortality in amphibians by interfering with electrolyte balance and osmoregulation [1], [2] and disrupting adaptive immune responses [3]. The disease has contributed to widely reported global amphibian population declines [4]–[7]. Yet, more than 15 years since its discovery, many aspects of the basic biology of the pathogen that causes the disease, the chytrid fungus *Batrachochytrium dendrobatidis* (denoted Bd), remain unknown. The pathogen's effects can be devastating, especially on its first contact with naïve host populations that may lack evolved defenses. As Bd is widely considered a primary cause of amphibian population declines, increasingly intensive research has focused on its emergence as a virulent pathogen, physiological tolerances, modes of transmission, and genetic and phylogeographic relationships among strains [3], [5], [8], [9].

Intervention strategies have been planned to protect native amphibian populations around the world [e.g. 10, 11], but their efficacy depends on rapid detection of the pathogen's first incursion into habitat occupied by at-risk species. As novel Bd strains now are being discovered, and Bd spreads through the international trade in amphibians [12], robust disease screening is essential to prevent further pathogen pollution [13]. Diagnostic assays that reliably detect the presence of Bd on infected animals, even at low infection intensity, are essential. Regions such as Madagascar that are home to a diverse collection of evolutionarily distinct endemic amphibians [14], [15] are of special concern [16], [17] and rapid responses are essential to prevent potentially large-scale species extinctions. More generally, conservation strategies, to be effective, must be built upon a foundation of robust research that employs reliable assay methods. Yet results of recent studies on Bd, even on fundamental issues (reviewed in [18]), vary widely. Erroneous inferences made on the infection status of populations only add to this problem.

**Figure 1:**
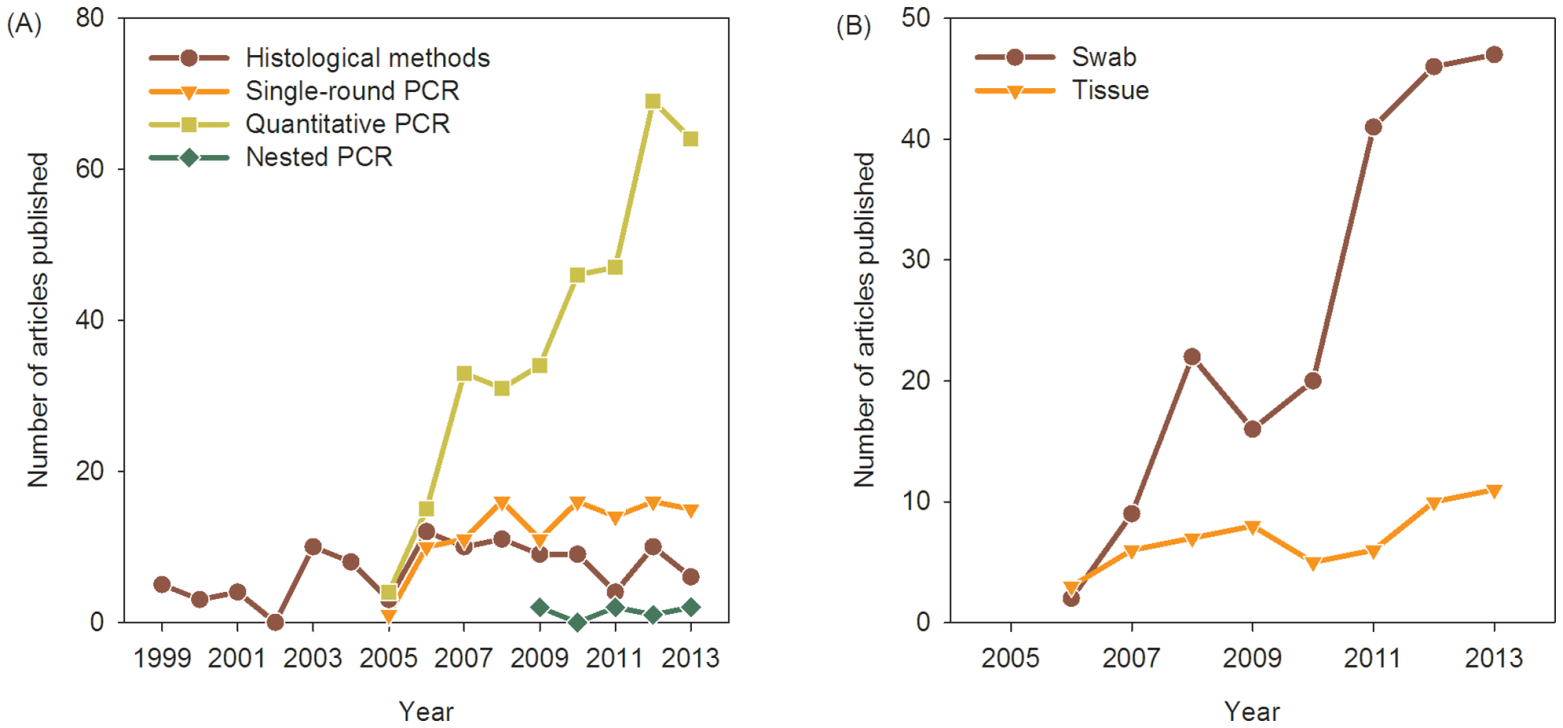
Bd diagnostic methods have changed over time. (A) Initial histological and immunohistological approaches rapidly have given way to PCR methods, especially qPCR. (B) At first, Bd was diagnosed by collecting toe-clips, sloughed skin, and other tissues but assaying Bd infection by swabbing began in 2006 and quickly became the predominant sampling method. Publication data from Science Citation Index, Zoological Record, and Google Scholar.

Initially, chytridiomycosis was diagnosed by histological [19] and immunohistological methods [20]. However, the procedures are time-consuming and correct interpretation very much depends on the quality of the tissue examined and the researchers' skills and training. Subsequently, Annis et al. [21] developed a PCR-based method and Boyle et al. [22] developed a quantitative TaqMan PCR assay. These methods detect Bd DNA quickly with very high sensitivity, making possible the rapid screening of large numbers of samples. Nested PCR can be even more sensitive in some circumstances, especially when working with with contaminated DNA or Bd strains with variable allele copy numbers [23], [24]. Nonetheless, qPCR remains the most common method for examining the presence of Bd in contemporary and historical samples (Figure 1).

Toe-clipping was the accepted sampling method for the detection of Bd until Hyatt et al. [25] recommended swabbing the skin of amphibians. Swabbing is viewed as equally sensitive but less invasive and logistically simpler than toe-clipping for Bd detection. Since then, the vast majority of Bd sampling has been done with swabs of the skin (Figure 1). However, extraction of DNA from swabs followed by PCR sometimes leads to inconsistent results. First, although researchers swab regions of the body most likely to be infected by Bd, some infected skin may be missed. Thus, some infections may escape detection, especially in individuals bearing low Bd loads, resulting in underestimation of Bd prevalence rates. Second, the distribution of Bd zoosporangia in the epidermis varies among species, with Bd colonizing only the superficial epidermis in some cases but penetrating into deeper skin layers in others [26]. Thus, the efficacy of DNA collection by swabs should vary in relation to differences among species in the pathogenesis of Bd as well as the extent of sloughing of infected tissue. Third, release of zoospores does not occur continuously but may vary in response to intrinsic factors or environmental triggers, further complicating the interpretation of DNA quantification from swabs. Fourth, contamination by environmental zoospores can lead to unreliable estimation of infection intensity by qPCR [27]. Because of these issues, estimates of zoospore genomic equivalents (ZGEs) based on swab sampling may be prone to error, especially when swabbing individuals with low Bd infection loads.

Throughout Asia, amphibians typically bear low Bd infection loads (Thailand [28], South Korea [29], India [30], Vietnam and Cambodia [31]; cf. Malaysia [32]). Bd appears to have been introduced only recently via the animal trade into countries that were thought to be Bd-free (e.g., Hong Kong [33], [34]; Singapore [35]), but large-scale die-offs have yet to be recorded. Studies on chytridiomycosis have focused on epizootics with dramatic incidents of morbidity and mortality, which may have created a sampling bias toward regions of the world with virulent strains of Bd [29], [36]. Because of their low infection loads, less virulent strains may be more difficult to detect and isolate [29].

Here we introduce a new Bd sampling procedure that overcomes difficulties with previous methods. Subjects are placed into containers where they release zoospores. The zoospores then are collected and Bd is extracted from filters for qPCR. We demonstrate the efficacy of this method in both laboratory and field settings. We then compare Bd diagnostic results obtained from qPCR runs on swabs and filtered water samples. Finally, we take repeated samples from the same subjects to investigate day-to-day variation in individuals' infection status.

## Materials and Methods

Experimental protocols were approved by the Institutional Animal Care and Use Committee (SNU-121210-2) and the Institutional Biosafety Committee (SNUIBC-P120725-2) of Seoul National University. Permits for fieldwork were issued by the mayors with jurisdiction over each locality (Hwacheon, Gangwon province; Pocheon, Gyeonggi province; and Jeonju, Jeollabuk province; see specific locality information below). The study species is not legally protected in South Korea.

### Animal sampling and husbandry

We collected 13 oriental fire-bellied toads (*Bombina orientalis*) during July and August 2012 from three localities in South Korea: Hwacheon (38°07′28.7″N 127°45′44.3″E) (n = 6), Pocheon (38°03′00.2″N 127°18′21.6″E) (n = 3) and Jeonju (35°47′03.5″N 127°08′29.6″E) (n = 4). We tested individuals for Bd infection by swab screening (see methods below) and found only one or two infected from each site. The frogs then were housed with others collected from the same locality in polypropylene tanks (w: 80 cm, l: 40 cm, h: 45 cm) for 6 months, during which time subjects cross-infected others in their tank [29].

Frogs were maintained at 20±2°C under a LD 12∶12 photoperiod. They were fed every second day with crickets (*Gryllus bimaculatus*) and mealworms (*Tenebrio molitor*). Water was changed weekly with fresh 0.5 µm-filtered tap water, which was first UV-sterilized and run through carbon filters. Medium-sized rocks were placed into the tanks to provide refuge and allow the frogs to emerge from the water. All individuals were photographed and identified by dorsal color patterns.

### Swabbing and filtering sampling methods

Each individual was tested for Bd infection using two different methods: swabbing and water filtering. Before swabbing, we rinsed each frog three times with filtered water to remove zoospores that may have been present from the environment. Then we swabbed the thighs, feet and ventral skin with 20 strokes each [25] using a sterile cotton swab (MW113, Medical Wire and Equipment, Corsham, Wiltshire, UK). Each subject was handled with a new pair of vinyl gloves to prevent cross-contamination among individuals [27]. Bd testing was conducted on all subjects using both methods 24 h and 16 days after the beginning of each trial.

After swabbing, each subject was placed into a polypropylene container (w: 9 cm, l: 15 cm, h: 8 cm) containing 150 mL of sterile water so that 90% of the frog's body was submerged to collect released zoospores. We chose 24 h as a collection period because this is the maximum time that zoospores have been observed to remain active [37]. After stirring the water to avoid settlement of the zoospores, 50 mL was collected from each container. The collected water was filtered repeatedly using a 5 mL syringe (Misosa, Ansan, South Korea) and 0.2 µm syringe filter (Puradisc 25, GE Healthcare, Giles, Buckinghamshire, UK). Alternatively, collected water can be filtered using sterile bottle top filters. We preferred the syringe filter option for easy transport to and from the field (see below).

To study zoospore release over time, 5 subjects were randomly selected and kept individually in separate containers for 5 days. The water was filtered every 24 h using the method described above. After each collection period, the subject was placed back into the container with 150 mL of fresh sterile water.

### Water filtering method in the field

In addition to our laboratory study, we conducted tests using similar methods in field conditions. We selected five sites to test the filtering method: Chuncheon (37°53′24.9″N 127°51′11.1″E) (n = 16), Hwacheon (38°07′28.7″N 127°45′44.3″E) (n = 16), Pocheon (38°03′00.2″N 127°18′21.6″E) (n = 28) and two sites in Yanggu (A: 38°14′03.8″N 128°02′23.6″E [n = 14]; B: 38°12′16.8″N 128°04′23.0″E [n = 18]). At each locality, we collected frogs and placed them into polypropylene tanks filled with 150 mL sterile ambient temperature water. We immersed subjects from Chuncheon for 24 h, but the others for 12 h. We filtered 50 mL water, stored the filters in dry ice, and transported them to the laboratory. The plastic tanks were sterilized with a 1∶20 diluted sodium hypochlorite solution and rinsed thoroughly with water after each collection. Other aspects of the procedures were identical to those used in the laboratory tests.

### DNA extraction

DNA from swabs was extracted using PrepMan Ultra (Applied Biosystems, Carlsbad, CA, USA) [22] to follow commonly used protocols (e.g. [35], [38], [39]). We added 50 µL PrepMan Ultra to each tube, which was heated for 10 min at 100°C. After cooling at room temperature for 3 min, tubes were centrifuged at 13,000 rpm for 3 min and the supernatant was collected.

To remove filter membranes from their plastic casing, we opened the syringe filters with pliers and then removed membranes with forceps. Pliers and forceps were flame-sterilized with 100% ethanol prior to use. DNA was extracted from the membrane of syringe filters with DNeasy blood and tissue kits (Qiagen, Valencia, CA, USA) using standard volumes of reagents following the manufacturer's instructions. All DNA samples were stored at −20°C. We used DNeasy for DNA extractions from filter membranes to decrease inhibitors [40].

To confirm that any differences observed between the results obtained with swabs and filters did not come about because of differences in extraction methods, we conducted an additional experiment on 7 individuals randomly selected among those used in the earlier study. The swabbing and filtering methods were identical with those described above. Each individual was swabbed using two swabs simultaneously and then was immersed for 24 h. One set of swab samples was extracted using DNeasy and the other with PrepMan Ultra. Similarly, two 50 mL volumes were filtered, each with new filters, one of which was used for DNA extraction with DNeasy and the other with PrepMan Ultra.

### Nested PCR assay

All collected swab and filter samples were tested for the presence of Bd using a highly sensitive nested PCR method targeting the 5.8S rDNA and ribosomal internal transcribed spacer regions (ITS) of Bd [23]. The first PCR was run in a volume of 20 µL containing 1 µL of DNA sample, 0.2 µM of forward primer Bd18SF1 (5′-TTTGTACACACCGCCCGTCGC-3′) and reverse primer Bd28SR1 (5′-ATATGCTTAAGTTCAGCGGG-3′), 0.2 mM of each dNTP, 2 mM of MgCl_2_ and 1.0 unit of Takara Ex Taq DNA polymerase (Takara Bio, Otsu, Shiga, Japan). The PCR conditions consisted of an initial denaturation at 94°C for 5 min, followed by 30 cycles of 30 s at 94°C, 30 s at 50°C, 2 min at 72°C, and a final extension at 72°C for 7 min.

A second PCR was run in a volume of 20 µL containing 1 µL of products from the first PCR, 0.2 µM of forward primer Bd1a (5′-CAGTGTGCCATATGTCACG-3′) and reverse primer Bd2a (5′-CATGGTTCATATCTGTCCAG-3′), 0.2 mM of each dNTP, 2 mM of MgCl_2_ and 1.0 unit of Takara Ex Taq DNA polymerase. The PCR conditions consisted of an initial denaturation at 94°C for 5 min, followed by 30 cycles of 45 s at 94°C, 45 s at 60°C, 60 s at 72°C, and a final extension at 72°C for 7 min. Each sample was run in duplicate together with positive (DNA from Bd culture) and negative (1 µL of ultrapure water) controls. Amplified PCR products were separated by agarose gel electrophoresis and visualized by ethidium bromide staining under UV light.

### Quantitative PCR assay

We conducted a qPCR assay [25] on an Applied Biosystems 7300 Fast Real-Time PCR system (Applied Biosystems, Carlsbad, CA, USA) to determine the presence of Bd and to estimate infection load from all swab and filter samples. Using qPCR, we also assessed the release cycle of Bd from 5 individuals over a 5-day sampling period. The primers and probe sequences for Bd detection and the procedures for qPCR followed Boyle et al. [22]: forward ITS1-3 Chytr (5′-CCTTGATATAATACAGTGTGCCATATGTC-3′), reverse 5.8S Chytr (5′-AGCCAAGAGATCCGTTGTCAA-3′), and the Chytr MGB2 probe (5′-6FAM CGAGTCGAACAAAT MGBNFQ-3′). PCR reactions were run in a 25 µL volume containing 5 µL of DNA sample, 0.9 mM of each primer, 0.25 mM of MGB probe, and 12.5 µL of Taqman Gene Expression Master Mix (Life Technologies). The PCR conditions consisted of an initial denaturation at 95°C for 10 min, followed by 50 cycles of 10 s at 95°C, and 1 min at 60°C. Each sample was assayed in duplicate together with standards of known Bd quantity (100, 10, 1, and 0.1 zoospores, strain AbercrombieNP-L.booroolongensis-09-LB-P7) and negative controls (5 µL ultrapure water). We estimated Bd zoospore genomic equivalents (ZGEs) from threshold cycle (Ct) values after corrections for dilution following DNA extraction and PCR procedures using SDS 1.2.3 software (Applied Biosystems).

To compare the efficacy of DNA extracted by DNeasy and PrepMan Ultra from swabs and filters, the presence of Bd was quantified using qPCR on an Illumina Eco Real-Time PCR system (Illumina, San Diego, CA, USA) in a volume of 10 µL containing 1x SYBR green quantitative PCR reagent kit (PhileKorea Technology, Seoul, South Korea), 0.25 mM of both ITS1-3 and 5.8S Chytr primers, and 2 µL of DNA. The PCR conditions consisted of an initial denaturation at 95°C for 10 min, followed by 50 cycles of 10 s at 95°C and 1 min at 58°C. In this experiment, infection intensity was estimated using a standard curve based on ITS copy numbers (3520, 352, 35.2, 3.52 ITS), which provides a more reliable measure than ZGEs when quantifying Bd strains with unknown numbers of ITS copies per zoospore [24].

ITS copies were obtained by amplifying the ITS region by PCR [21] using DNA from a Bd culture (strain AbercrombieNP-L.booroolongensis-09-LB-P7). Amplicons were purified and cloned using the RBC A&T cloning kit and accompanying HIT-DH5 alpha competent cells (RC001 and RH617, RBC Bioscience, Taipei, Taiwan) following the manufacturer's protocol. Plasmids that successfully inserted ITS PCR amplicons were extracted using a plasmid DNA extraction kit (Favorgen, Ping-Tung, Taiwan), and plasmid DNA was quantified using a NanoDrop 1000 spectrophotometer (Thermo Scientific, Wilmington, DE, USA) to calculate the number of ITS copies in the DNA extract.

Background signals of amplification, representing primer dimer artifacts, were obtained in some negative controls. Therefore, we added at least two controls to each run to define a threshold signal for comparison with each tested individual. If the highest value of qPCR Bd loads exceeded the highest value among negative controls, the subject was scored as Bd-positive. Similar procedures appear to be used in most studies employing qPCR to detect Bd, but details rarely are included in publications.

### Statistical analyses

Differences in estimates of infection intensity by swabbing and filtering were evaluated by Mann-Whitney U test. Bd prevalence was compared among field sites by Fisher's exact test. Bd ZGEs were compared within and among subjects and field sites by Kruskal-Wallis one-way analysis of variance. All statistical analyses were conducted with SAS 9.2 (SAS Institute, Cary, NC, USA) and inferences were drawn based on two-tailed probability distributions.

## Results

### False negatives in swab samples

Sampling methods differed in their qPCR results at both testing periods (Table 1). Subjects that were found to be infected by Bd based on water filter samples failed to test positive for Bd based on swabbing (subjects 4, 6, 7, 9 and 13 in the first test and 1, 3, 4, 6, 7, 9, 10, 11 and 12 in the second test, two weeks later).

**Table 1:** Infection status of subjects and prevalence estimates based on qPCR of DNA extracted from swabs and filtered zoospores taken at 24 h and 16 days.

Individual	1st sampling		2nd sampling	
	Swab	Filter	Swab	Filter
1	−	−	−	+
2	−	−	+	+
3	+	+	−	+
4	−	+	−	+
5	+	+	+	+
6	−	+	−	+
7	−	+	−	+
8	+	+	+	+
9	−	+	−	+
10	+	+	−	+
11	+	+	−	+
12	+	+	−	+
13	−	+	+	+
Prevalence	46%	85%	31%	100%

+ denotes subject tested Bd-positive.

− denotes subject tested Bd-negative.

**Figure 2:**
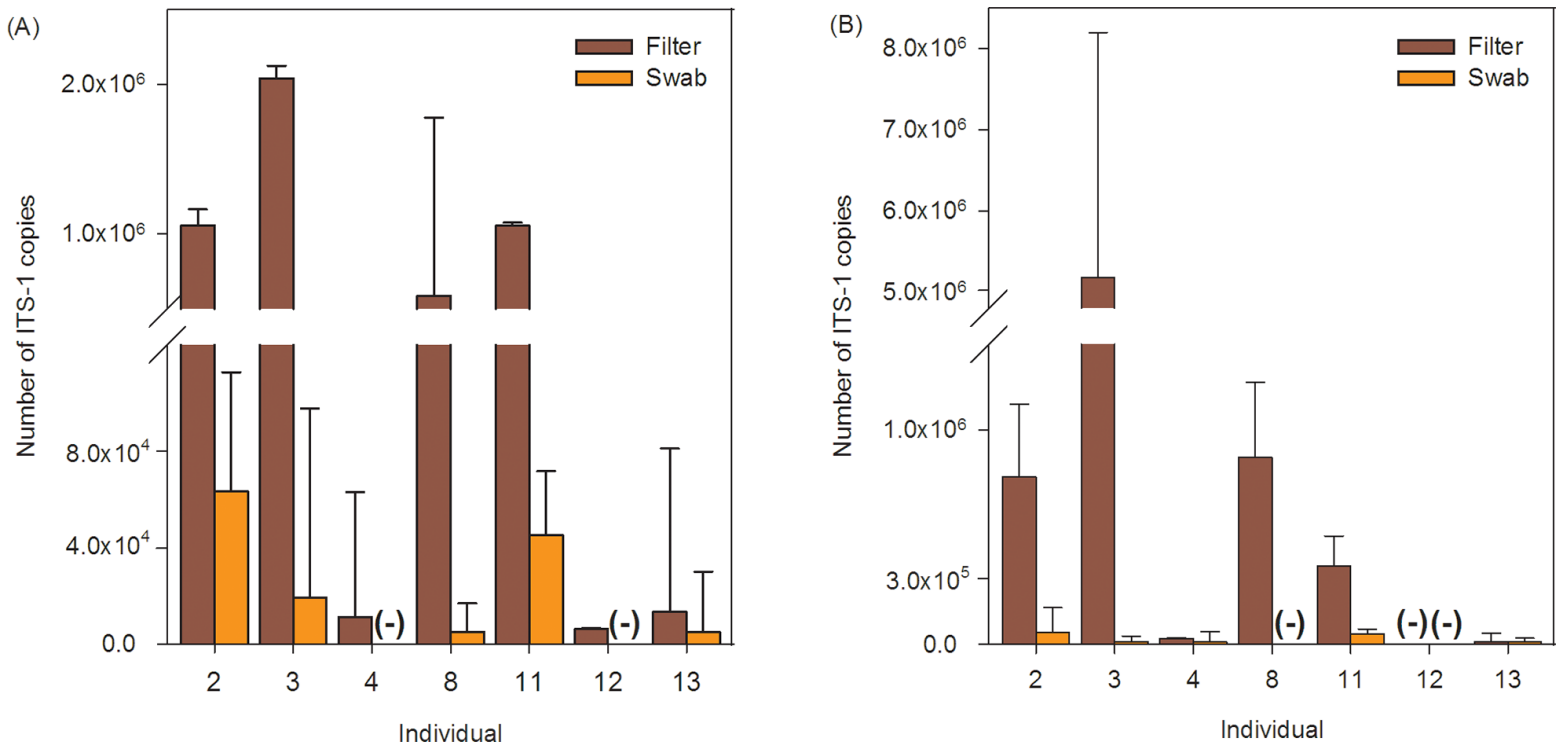
Number of ITS-1 copies collected by filters and swabs. DNA was extracted using DNeasy kits (A) and PrepMan Ultra (B) for both swabs and filters. (−) denotes negative Bd diagnosis. Error bars indicate 95% confidence limits. Ordinate scale differs below and above axis break.

Swab results varied with the extraction method used. Using DNeasy, all filter samples tested positive, but subjects 4 and 12 were falsely scored as negative based on swab samples (Figure 2A). With PrepMan Ultra, subject 8 falsely scored as negative using swabs (Figure 2B).

With swabs, results also varied based on the PCR method used (Table 2). For example, results of tests on subjects 2, 3 and 13 differed between qPCR and nested PCR in the second sampling period.

**Table 2:** Infection status of subjects dependent on PCR method of swab samples taken at 24 h and 16 days.

Individual	1st sampling		2nd sampling	
	NP^1^	TPA^2^	NP	TPA
1	−	−	−	−
2	−	−	−	+
3	+	+	+	−
4	−	−	−	−
5	+	+	+	+
6	−	−	−	−
7	−	−	−	−
8	+	+	+	+
9	−	−	−	−
10	+	+	−	−
11	+	+	−	−
12	+	+	−	−
13	−	−	−	+

1 Nested PCR.

2 Taqman probe assay.

### Estimating infection intensity

The infection loads estimated from swabs, 1.82±1.48 ZGEs (x ±SD; median: 1.37, range: 0.37–4.60 ZGEs), were significantly lower than those measured from filters, 2174.95±6540.54 ZGEs (median: 162.60, range: 12.60–41,370 ZGEs) (W = 66.0, z = 5.14, *P*<0.0001, Mann-Whitney U test, two-tailed; Figure S1).

The average number of ITS-1 copies detected by qPCR on filters (680,634±762,691) was approximately 24 times higher than that detected from swabs (28,815±27,064) taken from the same individuals using DNeasy (Figure 2A). When DNA was extracted using PrepMan Ultra, results were approximately 43 times higher for filters (1,206,808±1,979,989) than for swabs (28,816±27,064 (Figure 2B).

Up to five different ITS copies have been found in Korean Bd strains infecting *Bombina orientalis* [29]. Assuming that the same strains infected our subjects, we can estimate their average infection load to vary between 4,056 and 206,881 zoospores, notably higher than estimates based on traditional zoospore standards.

### Zoospore release cycle

**Figure 3:**
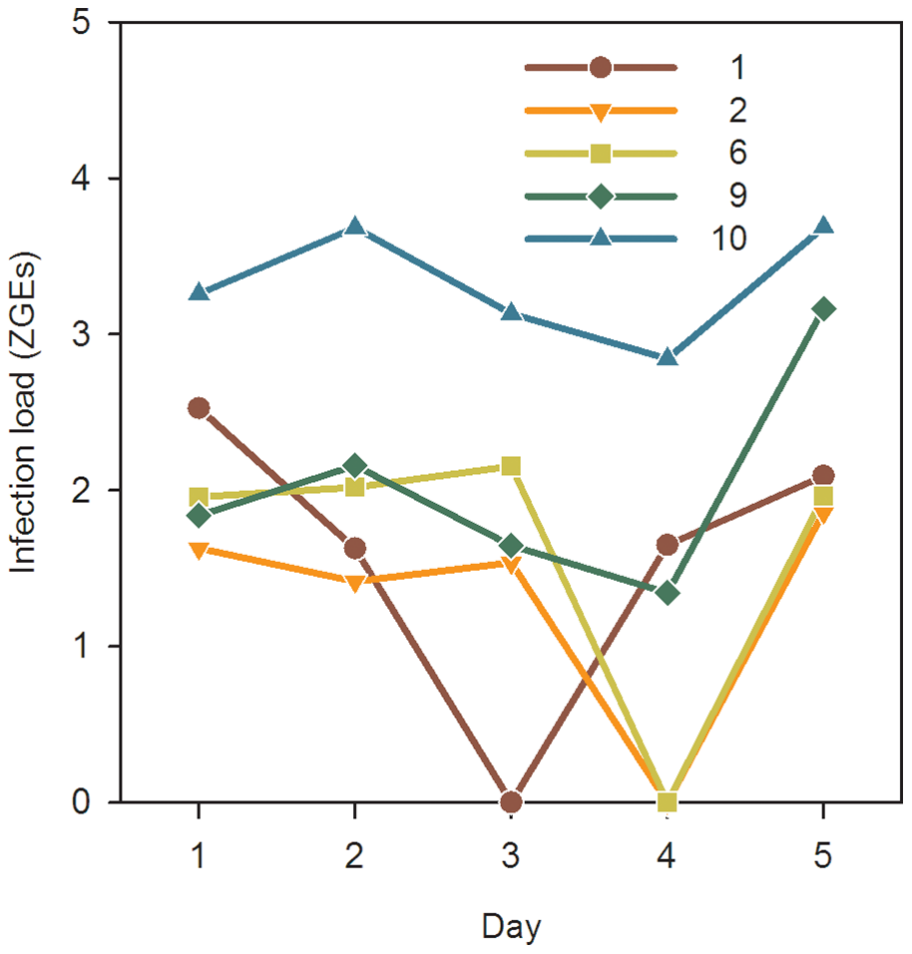
Zoospores released by infected subjects, represented as zoospore genomic equivalents (ZGEs), each day over a 5-day collection period. ZGEs are log-transformed.

Infected subjects discharged zoospores from zoosporangia intermittently over the 5-day experimental period, and individuals varied in their course of zoospore release (Figure 3). Overall, subjects significantly varied in the total number of zoospores released (χ^2^ = 12.817, 4 df, *P = *0.012, Kruskal-Wallis test, two-tailed). Subject 10 released the most zoospores during the experiment. No zoospores were detected in the water on day 3 (subject 1) nor day 4 (subjects 2 and 6), but on subsequent days these subjects tested positive again (Figure 3).

### Filtering in the field

Bd prevalence did not significantly vary among sites (Fisher's exact test, *P* = 0.95; Table 3). However, Bd prevalence values were somewhat higher than those previously measured in multi-species surveys across South Korea using swab samples [29]. The number of zoospores released did not significantly vary among sites (χ^2^ = 2.318, 4 df, *P* = 0.68, Kruskal-Wallis test, two-tailed) although immersion time was twice as long in Chuncheon as in the other two sites.

**Table 3:** Prevalence and infection intensity as determined in the field by zoospore filtering method.

Locality	Positive/Total	Prevalence (%)	Infection intensity (ZGE^1^ ± SD)
Chuncheon	3/16	18.8	66.23±21.68^2^
Hwacheon	3/16	18.8	49.63±14.86
Yanggu A	3/14	21.4	66.90±37.00
Yanggu B	4/18	22.2	45.22±15.12
Pocheon	8/28	28.6	80.20±51.67

As a comparison, a previous study on Korean amphibians by swabbing found a mean prevalence of 16.7% [29].

1 Zoospore genomic equivalents.

2 Chuncheon subjects were immersed for 24 h; all others for 12 h.

## Discussion

Diagnoses of amphibian chytridiomycosis today are based almost entirely on qPCR assays of swabbed skin. Consequently, our current understanding of the global distribution of Bd and its effects on host species is based largely on results of these assays. Despite its simplicity and sensitivity [22], [41]–[43], the method can yield unreliable results.

Our results call into question the accuracy of estimates of Bd prevalence, especially in regions around the world where epizootic chytridiomycosis has not been reported. In Asia, for example, Bd appears to be largely endemic and Bd loads are low [23], [29], [39]. Determinations based on swabs clearly underestimate Bd prevalence (Table 3). More worryingly, parts of Africa, Asia and Europe thought still to be Bd-free based on swab data [44], [45] already may harbor Bd. Consequent delays in implementation of intervention strategies to contain or eliminate the pathogen might lead to the extinction of endemic amphibian fauna in these regions.

DNA extracted from swabs can be amplified by several methods: standard PCR [21], qPCR [22] and nested PCR [23]. These methods are sensitive enough to detect one zoospore or even less. This acute sensitivity increases the risk of false positive results by cross-contamination [46] in the environment [27], [47], on the lab bench, or both. Thus, laboratories typically run repeated samples to control for inconsistent results. These problems, of course, are not particular to Bd screening, but best practice needs to be followed to ensure reproducibility among studies [48].

Quantitative PCR is quicker and more sensitive than histological analyses [27], and when done reliably can provide quantitative measures of infection intensity [41]–[43], but potential problems with swabbing have been largely overlooked until now. The accuracy with which infection status can be assessed reflects the efficacy with which swabbing correctly samples individuals' Bd loads. Our results suggest that swabbing often fails to detect infected individuals (Table 1, Figure 2A, B). Therefore, field studies using swabbing are likely to underestimate Bd prevalence (Table 3) and infection intensity (Figure 2). Because water filtration effectively samples the entire animal, the method we propose should provide more reliable prevalence estimates.

Problems can arise at several stages of sampling and processing to produce inaccurate results. Even using standardized procedures, picking up Bd DNA on subjects with low infection loads remains a stochastic process. A skilled histologist might need to spend hours examining many sections cut from several blocks before finding zoosporangia in the skin of an infected animal. Despite the increased sensitivity afforded by PCR, one never can be certain that infected tissues have been swabbed nor that DNA has been transferred to swabs. Bd zoosporangia are likely to be in layers of the stratum corneum that are frequently sloughed by infected individuals. Bd thus might not be detected on individuals that have sloughed prior to swabbing. Even if Bd DNA is picked up by swabs, samples may contain compounds that inhibit amplification [25], but DNA extracted from zoospores collected from subjects should have fewer contaminants and can be simpler to analyze.

Quantifying Bd infection loads from swab samples also can be problematic. The amount of Bd DNA collected on swabs depends on the condition of frogs' skin and how tissue is collected, both of which can result in inconsistent estimates of infection intensity by qPCR. The water filtering method collects zoospores released from infected subjects over time and thus can overcome these problems. We found that 30 to 50 times more DNA was extracted from zoospores collected from filter membranes than from swabs of the skin.

Methods of assessing Bd infection status of subjects by filtering water previously have been considered. Hyatt et al. [25] compared the efficacy of bathing, toe-clipping and swabbing as methods to detect Bd infection. Acknowledging that filtering might provide more reliable information on infection load, they nonetheless recommended swabbing as the standard Bd sampling method. Kirshtein et al. [49] used a filtering method to quantify environmental Bd zoospores from sediment and water. Hyman and Collins [50] tested a filtration-based pathogen monitoring system. Reeder, Pessier and Vredenburg [51] used a similar method to estimate infectivity of individuals, but with a short immersion time of 15 min that may be insufficient to detect low-level Bd infections.

Bd zoospores are released typically between four and five days after the maturation of zoosporangia [37]. The sharp drop in the number of zoospores detected in the water on days 3 and 4 of our experiment may be associated with this maturation cycle (Figure 3). Indeed, our results show that even after 24 hours, some subjects may not release zoospores. Therefore, individuals should be tested at least twice, several days apart, to be confident of their infection status.

Infection loads determined by filtering in field trials were lower than those found in well-controlled laboratory conditions (Table 3). Environmental factors such as temperature and pH may affect Bd activity and thus the results of assays [52]. Moreover, extended immersion times may be difficult to implement in large-scale projects or studies involving largely terrestrial species. To overcome these problems, additional studies on the release cycle of Bd are needed to optimize collecting conditions and immersion times. Although we used water filtration to sample individuals, this method also can serve to simultaneously sample many individuals. Water collected from amphibian habitat, or from containers holding amphibian shipments, can be tested quickly, efficiently, and inexpensively to assess the presence of Bd.

The worldwide distribution of the amphibian chytrid fungus is being compiled in the shared Global Bd Mapping Project database (www.bd-map.net [53]). These data are based almost entirely on qPCR analyses of DNA collected from swabbed animals. Our study suggests that the distribution maps may miss some regions that are infected by Bd, especially where infection loads are low. The methods we propose here for Bd sampling can assess infection status more precisely by directly targeting DNA of zoospores released from infected animals. Accurate assays of Bd infection status may prove crucial for the rapid implementation of intervention actions to protect global amphibian populations and control the spread of virulent Bd strains.

## Supporting Information

Figure S1 **Boxplot of zoospore genomic equivalents (ZGEs) as a function of Bd sampling method (n = 49 filter samples, 11 swab samples).** Median, interquartile range (box), and range (whiskers) are shown.
(TIF)
