# Supplementary figures and images for: Swabbing Often Fails to Detect Amphibian Chytridiomycosis under Conditions of Low Infection Load
Source: PLoS One. 2014 Oct 21;9(10):e111091. doi: 10.1371/journal.pone.0111091 (PMC4205094; doi:10.1371/journal.pone.0111091)

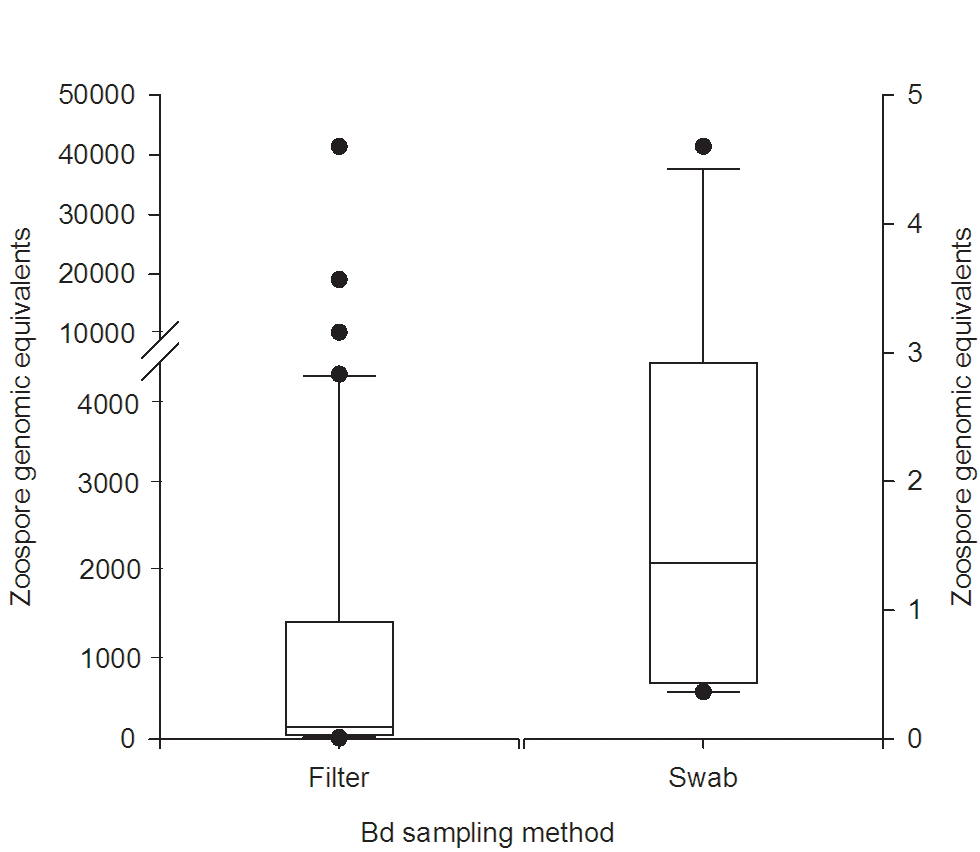

Supplement: Figure S1 — Boxplot of zoospore genomic equivalents (ZGEs) as a function of Bd sampling method (n = 49 filter samples, 11 swab samples). Median, interquartile range (box), and range (whiskers) are shown. (TIF) [file pone.0111091.s001.tif]
